# Bedside echocardiography in patients with suspected acute myocardial infarction without ST elevation

**DOI:** 10.1007/s00392-025-02724-2

**Published:** 2025-08-19

**Authors:** Paul Michael Haller, Alina Schock, Caroline Kellner, Jonas Lehmacher, Betül Toprak, Tau Sarra Hartikainen, Kira Marie Kiss, Gheas Alhaou, Kristin Peifer, Raphael Twerenbold, Tanja Zeller, Dirk Westermann, Johannes Tobias Neumann, Nils Arne Sörensen

**Affiliations:** 1 Department of Cardiology, University Heart and Vascular Center Hamburg, University Medical Center Hamburg-Eppendorf, Hamburg, Germany; 2 German Center for Cardiovascular Research (DZHK), Partner Site Hamburg/Kiel/Lübeck, Hamburg, Germany; 3 Department of Cardiology, University Heart Center Freiburg Bad Krotzingen, Bad Krotzingen, Germany; 4 University Center of Cardiovascular Science, University Heart and Vascular Center Hamburg, Hamburg, Germany; 5 Department of Epidemiology and Preventive Medicine, School of Public Health and Preventive Medicine, Monash University, Melbourne, Australia

**Keywords:** TTE, Acute myocardial infarction, Echocardiography, High-sensitivity cardiac troponin

## Abstract

**Background:**

Guidelines recommend using echocardiography in addition to high-sensitivity cardiac troponin (hs-cTn)–based assessment in patients with suspected acute myocardial infarction (MI), although data on its diagnostic and prognostic value is limited.

**Methods:**

Patients presenting with suspected MI without ST elevation to a tertiary center were enrolled. Final diagnoses were adjudicated by two cardiologists. Trained cardiology fellows conducted and interpreted bedside echocardiography in the emergency department as standard of care. Diagnostic performance measures (negative and positive predictive values [NPV, PPV] [95% confidence intervals]) to rule-in or rule-out MI were compared for patients with and without any pathological finding (any wall-motion abnormalities [WMA] or systolic left ventricular dysfunction) in patients assigned to the observe group by a 0/1-hs-cTnI-algorithm. Cox regression analysis was conducted to investigate the association of pathological findings with all-cause death.

**Results:**

Overall, 2779 patients with available echocardiography were included (median age 63 years, 366 [13.2%] with MI, 2158 [77.6%] with data on WMA, median follow-up 5.2 years). The PPV to identify MI using bedside echocardiography in the observe group was low (PPV 8.9 [6.3, 12.5]), and the NPV was 95.6 [93.9, 96.9]. After adjustment for relevant covariates, patients with any compared to no pathological finding  were at increased risk for all-cause death during follow-up (HR 1.36 [1.06, 1.74], *p* = 0.014).

**Conclusions:**

In this observational study, it could not be shown that bedside echocardiography in the context of suspected acute MI without ST elevation substantially improves diagnostic accuracy beyond hs-cTn-based diagnostic protocols to identify MI in patients assigned to the observe zone. However, findings on echocardiography helped to identify high-risk patients. More prospective evidence on the use of bedside echocardiography is needed to increase the body of evidence.

Trial registration

ClinicalTrials.gov Identifier: NCT02355457.

**Graphical Abstract:**

**Figure:**
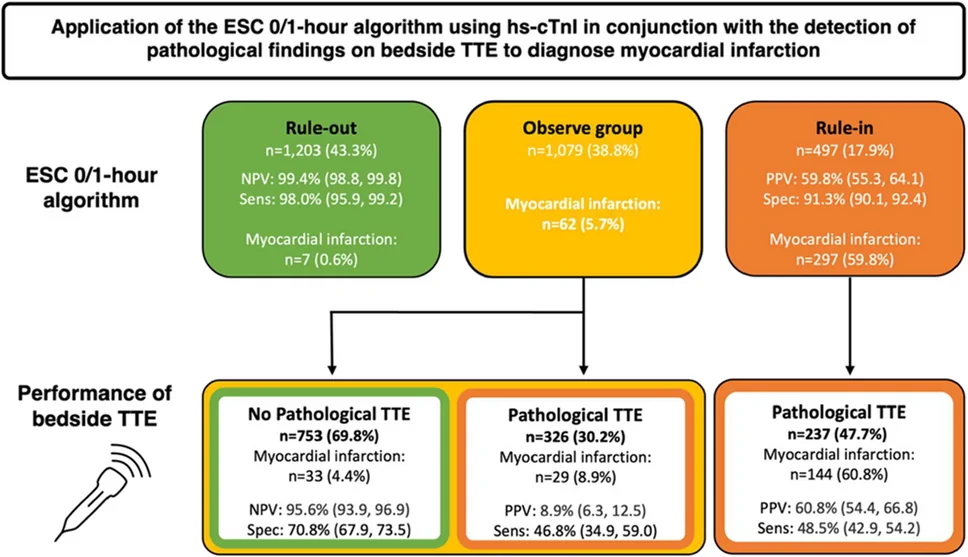

**Supplementary Information:**

The online version contains supplementary material available at https://doi.org/10.1007/s00392-025-02724-2.

## Introduction

The diagnostic evaluation of patients with suspected myocardial infarction (MI) relies on the assessment of symptoms, findings on 12-lead electrocardiograms, and the evaluation of cardiac troponin. Accelerated diagnostic protocols based on high-sensitivity cardiac troponin assays (hs-cTn) facilitate a rapid rule-out of MI for approximately half of patients and identify those at high risk for MI, usually within one or 2 hours (h) [1–4].

Still, between 25 and 45% of patients remain in an intermediate-risk group (also known as the “observe” group), requiring further diagnostic work-up [3–5]. In addition to further serial determination of hs-cTn after 3 h, European and US guidelines also advocate the conduction of transthoracal echocardiography (TTE) in specific settings[5, 6]. However, data on the diagnostic performance of TTE in patients with suspected MI are scarce. A previous study of only 100 patients reported limited diagnostic value by the assessment of regional wall-motion abnormalities (WMAs) to identify or exclude acute MI in patients presenting with symptoms of MI and concomitant left bundle branch block [7]. However, a large-scale evaluation of the diagnostic value of bedside TTE in this setting has not been performed yet.

To address this clinical need, the aim of this study was to assess the diagnostic performance and prognostic value of bedside TTE in patients presenting with suspected acute MI. We report performance parameters of bedside TTE to rule out or rule in MI, focusing on intermediate-risk patients, in whom first hs-cTn results are inconclusive, and investigated the value of bedside TTE for risk stratification of patients for clinically important events during long-term follow-up.

## Methods

### Study population

The current study used data from the Biomarkers in Acute Cardiac Care (BACC) cohort study (Supplementary Table 1), which is a single-center, observational study of prospectively enrolled patients with symptoms suggestive of acute coronary syndrome presenting to the emergency department of the University Medical Center Hamburg-Eppendorf. Patient enrolment started in 2013 and is ongoing. For this analysis, all patients enrolled up to May 2022 were included, excluding those with a final diagnosis of ST-elevation myocardial infarction.

All patients provided written informed consent, the study was conducted according to the Declaration of Helsinki and was approved by the appropriate ethics committee. The BACC cohort study is registered with clinicaltrials.gov (NCT02355457). Detailed descriptions of the study have been published previously [3, 4, 8–12].

### Clinical management

The clinical management of patients was fully at the discretion of the physician in charge. Diagnostic and therapeutic management followed institutional standards and respective guidelines as applicable [1, 13]. This included assessment of symptoms, routine recordings of a 12-lead ECG at presentation and after 3 h, routine laboratory diagnostics, and imaging diagnostics as needed. For the evaluation of suspected MI, serial hs-cTnT (Elecsys, Roche Diagnostics, Basel, Switzerland; up to May 2020) or serial hs-cTnI (Atellica IM, Siemens, Erlangen, Germany; from June 2020 onwards) concentrations at presentation and after one and 3 h were routinely determined in the central laboratory.

### Bedside echocardiography

The conduction of bedside TTE in the Emergency Department as part of the diagnostic evaluation for suspected MI was not mandated by institutional standard operating procedures but was encouraged in all patients. A Vivid S5 or S6 and later S70N (GE Healthcare, Chicago, IL, USA) ultrasound machine equipped with a M5SC series probe was used. For the purpose of the BACC study, the left ventricular function (categorized into normal, mildly, moderately, or severely impaired), the presence of regional WMAs, and the identification of severe valvular pathologies have been recorded. Per institutional standard, information on WMA were only reported if image quality and acoustic window allowed a reasonable assessment. In case of uncertainties, the echocardiography lab and/or the cardiology attending are routinely consulted. All echocardiographic evaluations were performed outside of the BACC study and served solely for the clinical management of patients. The timing of bedside TTE was at the discretion of the treating physician and could happen during or after determination/reporting of hs-cTn. Only bedside TTE data recorded during the patient’s stay in the chest pain unit were considered for the present analysis. If patients were admitted and underwent TTE in the echocardiography lab again, the latter data were not considered for the present analysis.

### Study-specific evaluation

Biobanking of serum and plasma samples was performed at baseline and after 1 and 3 h. All demographic and clinical data were obtained by patient interview and medical chart abstraction using pre-specified case report forms.

The final diagnosis for each patient was adjudicated according to the Fourth Universal Definition of MI [14] using all available information from the medical records, study case report forms, and hs-cTnT. Adjudication was conducted by two independent cardiologists at least 1 month after the patient’s index presentation, with disagreement resolved by assessment of a third cardiologists. In patients who were clinically managed using hs-cTnI, hs-cTnT was available for deriving the final diagnosis using research samples.

Yearly, census follow-up was carried out by trained study personnel who contacted patients, their relatives, and (if needed) family physicians to conduct a structured interview on pre-specified events. These included all-cause death, acute MI, myocardial revascularization, and hospitalization for cardiac reasons. As needed, reports from hospitals or ambulatory care facilities were retrieved from patients, relatives, or family physicians, and relevant information was extracted. In case no telephone contact has been established, patients were contacted by mail and email. Lastly, the local death registry was consulted to assess potential  death and date of death. The overall proportion of lost-to-follow-up for the endpoint of all-cause death was < 1% for the overall study population.

### Statistics

Our primary aim was to investigate the diagnostic performance of bedside TTE for the evaluation of patients with suspected MI and without ST elevation, wherefore all patients with ST-elevation myocardial infarction were excluded from the analyses. Continuous variables are provided as median (with 25th and 75th percentile), and categorical variables as number (percentages). Differences between groups were given as absolute standardized difference (95% confidence interval [CI]) and calculated using Bootstrap with 6000 replications for continuous variables and using the chi-square test for binary variables. The median follow-up time was estimated by the reverse Kaplan–Meier estimator.

We investigated the diagnostic performance of any versus no pathological finding, which was defined as any WMA (regional hypokinesia or akinesia) or the presence of systolic left ventricular (LV) dysfunction (defined as LV ejection fraction < 50%) detected by bedside TTE. If WMA were not reported due to an impaired acoustic window, only the assessment of LV function was considered for the present purpose. Performance parameters of interest included sensitivity, specificity, negative predictive value (NPV), and positive predictive value (PPV) (all reported as % [95% confidence interval]) and were calculated for no versus any pathological finding to rule-in or rule-out MI. Evaluation was performed after application of the ESC 0/1-h algorithm using a hs-cTnI assay (ARCHITECT, Abbott Diagnostics, Chicago, IL, USA) in the observe- and rule-in groups, respectively.

For the prognostic assessment, all-cause death was investigated as the primary endpoint, and a composite of all-cause death, incidental MI, unplanned myocardial revascularization, and hospitalization for cardiac reasons as a secondary endpoint. For crude comparison of groups, we provide Kaplan–Meier plots and compare differences through 5 years using the log-rank test. Cox-proportional hazard models adjusted for diabetes, hypertension, family history of coronary artery disease, prevalent coronary artery disease, history of stroke, congestive heart failure (all binary), age (in years), sex (male versus female), and baseline hs-TnI (absolute concentration) were performed. All statistical analyses were performed using R version 4.3.1 (R Foundation for Statistical Computing) [15].

## Results

Among 3344 patients, data on TTE were available in 2779 (83.1%), and for 2158 (77.7%) patients, data on regional WMA were quantifiable as well. Supplementary Table 2 summarizes baseline characteristics of patients with and without information on TTE. Overall, Type 1 MI (T1MI) (9.0% vs. 5.4%) and unstable angina pectoris (8.4% vs. 3.8%) were more common in patients receiving bedside TTE, whereas chronic myocardial injury was less common (24.2% vs. 34.5%). More detailed clinical reasons for the final diagnoses, along with findings of bedside TTE, are provided in Supplementary Tables 3 and 4.

In the study population of 2779 patients, the median age was 63 (51, 75) years, and 1774 (63.8%) were male. The final diagnoses were T1MI in 253 (9.1%), type 2 non-ST-elevation MI (T2MI) in 113 (4.1%), acute myocardial injury in 82 (3.0%), chronic myocardial injury in 669 (24.1%), and other diagnoses in 1662 (59.8%). Cardiovascular risk factors were frequent and more prevalent in patients with any pathological finding (i.e., reduced LV function and/or WMA), with details provided in Table 1.

**Table 1:** Patient characteristics

	All (*N* = 2779)	No pathological findings (*N* = 2128)	Any pathological finding (*N* = 651)	Absolute standardized difference (95% CI)
Age (years)	63 (51, 75)	60 (49, 73)	71 (59, 79)	11 (7, 11)
Male	1774 (63.8)	1298 (61.0)	476 (73.1)	11.68 (7.6, 15.7)
Body mass index (kg/m^2^)	26.0 (23.2, 29.0)	26.0 (23.0, 29.0)	27.0 (24.0, 30.0)	1 (0, 1)
Hypertension	1761 (63.5)	1245 (58.6)	516 (79.5)	21.2 (17.4, 25.1)
Hyperlipoproteinemia	908 (32.7)	595 (28.0)	313 (48.1)	20.1 (15.7, 24.4)
Current smoker	591 (21.8)	449 (21.6)	142 (22.6)	0.9 (− 2.9, 4.7)
Former smoker	603 (22.3)	439 (21.1)	164 (26.1)	4.7 (0.7, 8.6)
Diabetes	348 (12.6)	207 (9.8)	141 (21.9)	12.2 (8.6, 15.7)
Family history of coronary artery disease	519 (19.7)	407 (20.2)	112 (18.1)	− 2.3 (− 5.8, 1.3)
History of coronary artery disease	924 (33.2)	542 (25.5)	382 (58.7)	33.3 (29.1, 37.6)
Stroke	150 (5.4)	101 (4.8)	49 (7.5)	3.2 (0.8, 5.5)
Atrial fibrillation	330 (11.9)	184 (8.7)	146 (22.4)	13.8 (10.3, 17.3)
Previous myocardial infarction	470 (16.9)	225 (10.6)	245 (37.8)	27.6 (23.6, 31.6)
Ischemic signs on ECG	547 (20.1)	299 (14.3)	248 (39.5)	25.1 (20.9, 29.3)
Coronary angiography during index presentation	682 (24.5)	363 (17.1)	319 (49.0)	31.8 (27.6, 36)
Revascularization during index presentation	352 (12.7)	171 (8.0)	181 (27.8)	19.5 (15.8, 23.2)
hs-cTnI 0 h (ng/L)	5.3 (2.6, 15.5)	4.1 (2.2, 9.1)	19.1 (7.5, 92.8)	15.8 (11.8, 21.7)
hs-cTnI absolute delta 1 h (ng/L)	0.1 (− 0.7, 1.2)	0.1 (− 0.5, 1.0)	0.1 (− 1.9, 3.6)	0 (− 0.1, 0.4)
eGFR (mL/min/1.73 m^2^)	79 (60, 93)	82 (65, 95)	64 (46, 83)	− 18 (− 22, − 15)

Variables are provided as *N* (%) or median (25th, 75th percentile). Any pathological findings include systolic left ventricular dysfunction or the presence of any regional wall-motion abnormality, being either hypokinesia or akinesia. The absolute standardized difference is provided with its 95% confidence interval (CI), calculated using bootstrap with 6000 replications and comparing patients with and without any pathological finding

*ECG* electrocardiogram, *eGFR* estimated glomerular filtration rate, *hs-cTnI* high-sensitivity cardiac troponin I

In patients with available TTE data, the median follow-up was 5.17 years (max follow-up 9.73 years), during which 389 death events and 969 MACE events were recorded.

### Diagnostic performance of bedside TTE following the ESC 0/1-h algorithm

**Fig. 1:**
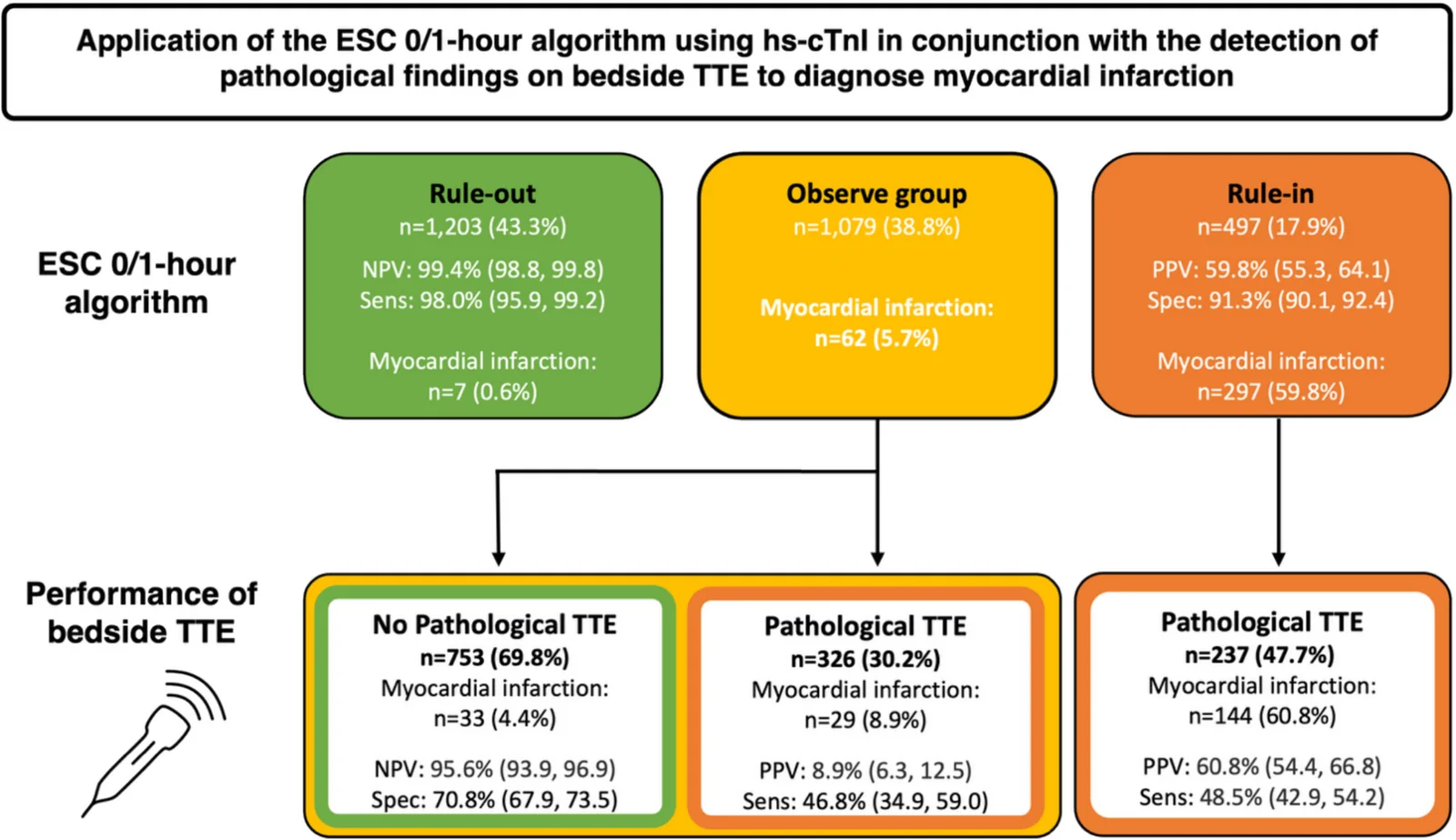
Diagnostic performance of bedside TTE in patients with suspected MI without ST elevation. In the upper part, the performance of the European Society of Cardiology (ESC) 0/1-hour algorithm using hs-cTnI is shown for a population of patients with suspected MI without ST elevation. Additionally, the performance of bedside TTE has been investigated in the observe and rule-in groups after application of the ESC 0/1-hour algorithm. Here, the detection of any pathological finding, being either any kind of wall-motion abnormality (hypokinesia or akinesia) or systolic left ventricular impairment, was investigated to rule-out or rule-in MI. The performance of TTE was not tested in the rule-out group. ESC, European Society of Cardiology; hs-cTnI, high-sensitivity cardiac troponin I; MI, myocardial infarction; NPV, negative predictive value; PPV, positive predictive value; TTE, transthoracic echocardiography

The ESC 0/1-h algorithm using hs-cTnI was applied, assigning 1203 (43.3%) patients to the rule-out group, 497 (17.2%) to the rule-in group, and 1079 (38.8%) remained in the observe group (Fig. 1). The additional diagnostic value of bedside TTE was tested in patients of the observe and rule-in groups.

Of 1079 patients assigned to the observe group, 62 patients had a final diagnosis of MI (5.7%). Using TTE, 29 patients with MI were correctly identified, whereas 33 patients would have been missed (Fig. 1, Table 2). Furthermore, in 720 patients, MI was correctly ruled out using bedside TTE; however, 297 patients had pathological findings but no MI (false positive). Detailed results for the diagnostic performance are provided in Fig. 1 and Table 2.

**Table 2:** Performance of bedside transthoracic echocardiography to rule-in or rule-out acute myocardial infarction

	*N*	Sensitivity	Specificity	PPV	NPV	TP	TN	FN	FP
ESC 0/1-h observe	1079	46.8 (34.9, 59.0)	70.8 (67.9, 73.5)	8.9 (6.3, 12.5)	95.6 (93.9, 96.9)	29	720	33	297
ESC 0/1-h rule-in	497	48.5 (42.9, 54.2)	53.5 (46.6, 60.3)	60.8 (54.4, 66.8)	41.2 (35.3, 47.2)	144	107	153	93

Performance parameters are calculated for the presence of any pathological finding, being any regional wall-motion abnormality (hypokinesia or akinesia) or any systolic left ventricular dysfunction detected by bedside transthoracic ultrasound

*FN* false negative, *ESC* European Society of Cardiology, *FP* false positive, hs-cTnI high-sensitivity cardiac troponin I, *NPV* negative predictive value, *PPV* positive predictive value, *TN* true negative, *TP* true positive

Additionally, we tested the performance of bedside TTE in 497 patients assigned to the rule-in group. Here, pathological findings of TTE were not associated with greater identification of MI beyond the 0/1-h algorithm (PPV 60.8% [54.4, 66.8] versus 59.8% (55.3, 64.1), *p* = 0.67; Fig. 1, Table 2).

As part of a sensitivity analysis, we also investigated the diagnostic utility of identified WMA alone. Detailed results are provided in Supplementary Table 5, with a non-significant increase of the PPV in the rule-in group.

### Findings in bedside TTE and clinical events

**Fig. 2:**
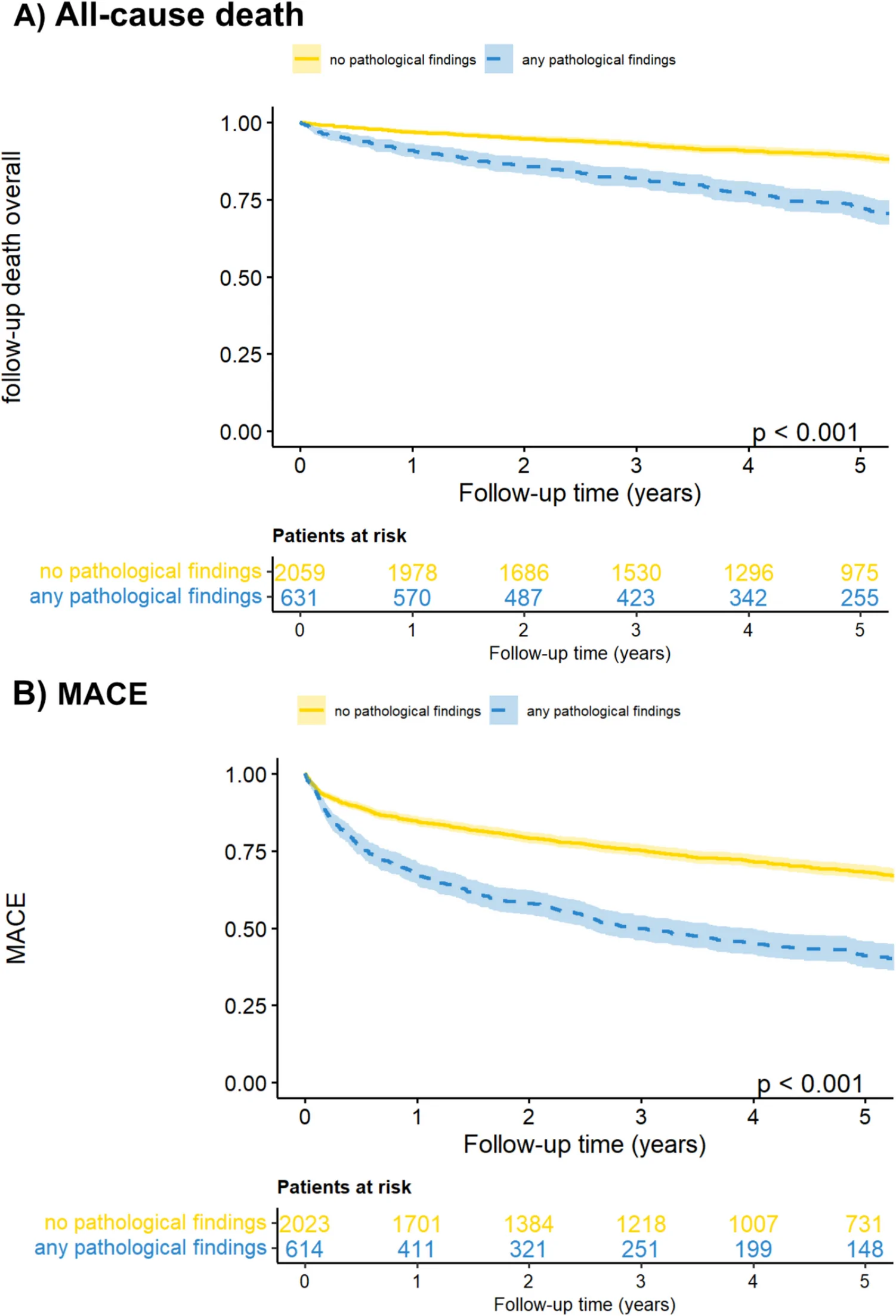
All-cause death (**A**) and MACE (**B**) stratified by any pathological finding on bedside transthoracic ultrasound. MACE includes the endpoints all-cause death, incident myocardial infarction, unplanned myocardial revascularization, and cardiac hospitalization. Shaded areas provide 95% confidence intervals. *p* Values were calculated using the log-rank test at 5 years

Additionally, we determined whether findings on bedside TTE were useful for risk stratification of patients with respect to future events. Patients with any versus no pathological finding had a significantly higher rate of all-cause death and MACE (including all-cause death, unplanned myocardial revascularization, hospitalization for cardiac reasons, or incidental MI) through 5 years (*p* < 0.001 for both, Fig. 2A, B). Upon full adjustment, any compared to no pathological finding was significantly associated with all-cause death (adj-HR 1.36 [1.06, 1.74], *p* = 0.014), but not MACE (adj-HR 1.16 [0.98, 1.36], *p* = 0.08).

**Fig. 3:**
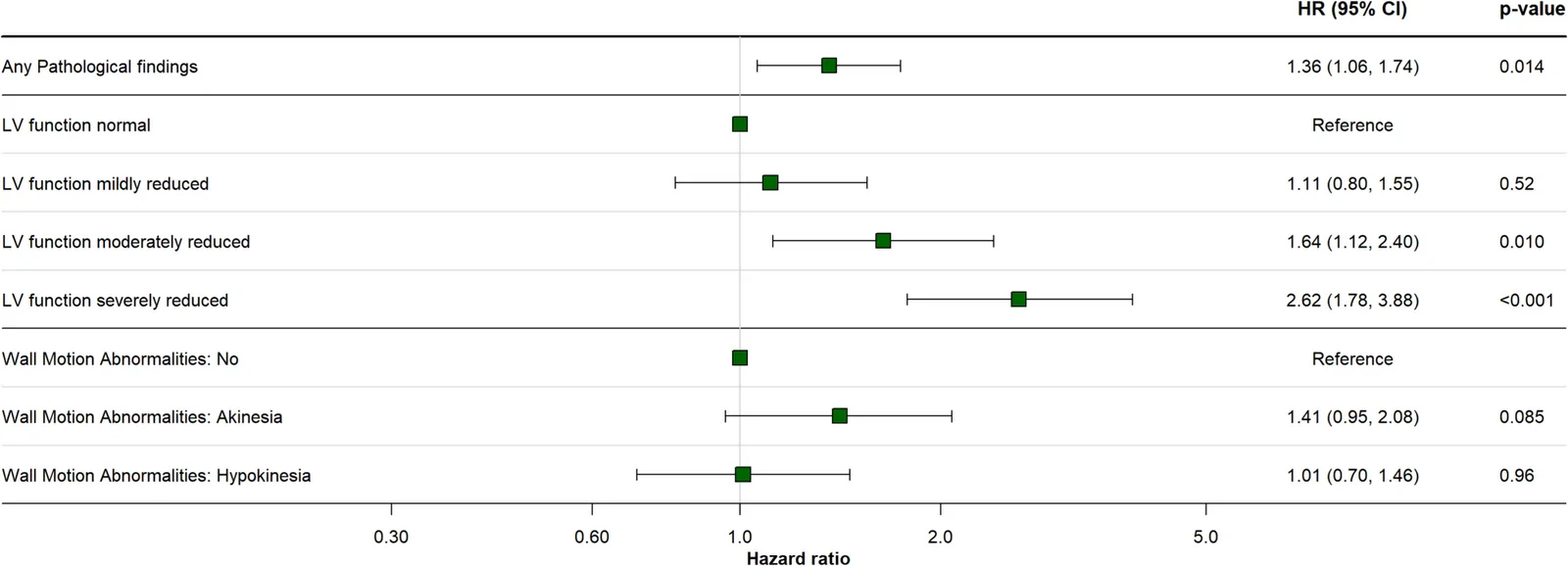
Adjusted hazard ratios (95% confidence intervals) of pathological findings on bedside transthoracic echocardiography for all-cause death. This figure shows results for the individual findings on bedside transthoracic echocardiography derived from three dedicated models, and their adjusted risk for all-cause death is shown. Adjustment was made for age, sex, diabetes, hypertension, family history of coronary artery disease, prevalent coronary artery disease, history of stroke, congestive heart failure, and baseline high-sensitivity cardiac troponin I

Figure 3 provides the individual findings on bedside TTE and their association with all-cause death adjusted for relevant covariates. Overall, moderate (adj-HR 1.64 [1.12, 2.40]) or severe impairment (adj-HR 2.62 [1.78, 3.88]) of systolic left ventricular function were independently associated with all-cause death.

## Discussion

The present study is the first large-scale analysis of the diagnostic performance of bedside TTE for suspected acute non-ST-elevation MI. It could not be shown that information on LV function or on WMA in the context of suspected acute MI without ST elevation substantially improves diagnostic accuracy beyond hs-cTn-based diagnostic protocols to identify MI. However, it provides important information on the patient’s short- and long-term risk of future cardiovascular events and death.

In the setting of suspected MI, the diagnostic management relies on the assessment of symptoms, recording of an ECG, and, in those without ST elevation, on the assessment of hs-cTn. Additionally, European and American guidelines recommend further examination using echocardiography (class I, level of evidence rating “C”), particularly for the assessment of differential diagnoses, in patients with signs of heart failure, and in those being assigned to the observe/intermediate-risk group [1, 6]. Bedside TTE may be conducted to evaluate cardiac function and wall motion, treating abnormalities of the latter as a surrogate for (regional) ischemia and, thus, infarction. While this is fundamental to the way we understand the pathophysiology of MI, the additional benefit beyond biomarker-based protocols has not been investigated, and as such, the benefit also specifically in intermediate-risk patients remains unclear.

In the present study, the diagnostic utility of an assessment of regional WMA and systolic LV function in patients with suspected acute MI without ST elevation is investigated after patients are stratified using a hs-cTn-based rapid diagnostic protocol. The additional use of bedside TTE might be particularly helpful in the “observe” group. In these patients, the echocardiographic assessment could aid physicians in the decision to rule-out or rule-in MI, as the serial determination of hs-cTn was inconclusive. However, the identification of regional WMA or systolic LV dysfunction in patients assigned to the observe group had a low positive predictive value and sensitivity to identify patients with MI. In 1079 patients assigned to the observe group, 29 patients have been identified correctly by TTE; however, 33 have not, resulting in a very low PPV and sensitivity. With respect to the rule-out of MI, the finding of a normal TTE evaluation resulted in a NPV of 95.6%, and specificity was 70.8%. Overall, 33 patients with MI would not have been identified by TTE assessment in the observe group. While we investigated a combined endpoint of “any pathological findings,” including systolic LV impairment and any WMA, our findings were similar in a sensitivity analysis only considering WMA. Still, these results suggest that ruling out pathological findings may help in the work-up of patients in the observe group to rule-out MI.

While the utility of bedside TTE may not be overt on top of hs-cTn for diagnostic purposes with respect to suspected MI without ST elevation, it may still be very helpful for risk stratification. Pathological findings on bedside TTE were independently associated with prognosis. The recent expert consensus decision pathway by the American College of Cardiology [6] explicitly recommends a TTE examination for the evaluation of structural abnormalities in patients with T2MI, and those with acute or chronic myocardial injury, respectively. A basic echocardiographic assessment allows identification of exactly those patients at high risk for death and cardiovascular events. Of note, this association was independent from baseline hs-cTnI, further highlighting its utility for risk stratification. Altogether, this strengthens the recommendation to perform TTE in selected patients deemed at intermediate or high risk.

Patients in the observe group frequently require further diagnostic workup [1, 8, 9]. Previously, Lopez-Ayala et al. reported modified hs-cTn cut-offs for application in the observe group after 3 h, aiming to refine and improve the diagnostic management after application of a 0/1-h algorithm [16]. Although the diagnostic performance improved by this approach, only one third of all patients initially assigned to the observe group were hereby assigned to the rule-out or rule-in groups, respectively, resulting in a considerable proportion of patients still without clear assignment after 3 h.

### Strengths and limitations

The present analysis is based on a large cohort of patients presenting to the emergency department of a tertiary hospital with suspected acute MI. There was no study-specific guidance to conduct TTE. The decision to conduct an echocardiographic assessment was fully at the discretion of the physician in charge, although per institutional standards, the conduction is encouraged in most patients, which is reflected by a high proportion of patients receiving TTE (~ 85%). While patient characteristics appear broadly similar comparing patients with and without TTE, still, selection bias cannot be ruled out entirely. The TTE was conducted and interpreted by cardiology fellows in the emergency department. Settings in which a bedside TTE is conducted, operator skills and technical circumstances may have influenced our results. Differential diagnoses for which an echocardiographic assessment might be useful have not been systematically recorded, preventing dedicated analyses. Also, due to the unavailability of multiple echocardiography assessments during the acute presentation and the retrospective nature of the present study, calculations on intra- and interobserver bias were not possible. Measures of diastolic dysfunction or strain analyses are not routinely assessed in the Emergency Department and, thus, have not been collected as part of the BACC study. The final diagnoses were adjudicated according to the 4th Universal Definition of MI by at least two independent cardiologists evaluating all available information from electronic patient records. Albeit this practice is common for this kind of observational study, there remains a small chance for misclassification due to the unavailability of a gold standard for the diagnosis of MI apart from cardiac troponin.

## Conclusion

In this observational analysis, it could not be shown that information on LV function or on WMA in the context of suspected acute MI without ST elevation substantially improves diagnostic accuracy beyond hs-cTn-based diagnostic protocols to identify MI in patients assigned to the observe group. Findings on TTE help to identify patients at risk for clinically important events, underscoring a high value for risk assessment in the emergency department.

## Supplementary Information

Below is the link to the electronic supplementary material.

Supplementary file1 (DOCX 71 KB)

## Data Availability

The data that support the findings of this study are available on request to the corresponding author for qualified researchers. The data are not publicly available.
