# Supplementary material for: Bedside echocardiography in patients with suspected acute myocardial infarction without ST elevation
Source: Clin Res Cardiol. 2025 Aug 19;115(12):2152–60. doi: 10.1007/s00392-025-02724-2 (PMC13751795; doi:10.1007/s00392-025-02724-2)
Supplement: Supplementary file 1 [file 392_2025_2724_MOESM1_ESM.docx]

**Supplementary material for**

**“Bedside echocardiography in patients with suspected acute myocardial infarction without ST-elevation”**

Paul M Haller, MD, PhD^1,2^; Alina Schock, MD^1^; Caroline Kellner, MSc^1^, Jonas Lehmacher, MD^1^; Betül Toprak, MD^1,2^; Tau S Hartikainen^,^ MD^3^; ; Kira M. Kiss^1^, Gheas Alhaou^1^, Kristin Peifer^1^, Raphael Twerenbold, MD^1,2,4^; Tanja Zeller, PhD^1,2,4^; Dirk Westermann, MD^1,2,3^; Johannes T Neumann, MD, MCR^1,2,5^, Nils A Sörensen, MD^1,2^

1) Department of Cardiology, University Heart and Vascular Center Hamburg, Hamburg, Germany.

2) German Center for Cardiovascular Research (DZHK), Partner Site Hamburg/Kiel/Lübeck, Hamburg, Germany.

3) Department of Cardiology, University Heart Center Freiburg Bad Krotzingen, Bad Krotzingen, Germany

4) University Center of Cardiovascular Science, University Heart and Vascular Center Hamburg, Hamburg, Germany.

5) Department of Epidemiology and Preventive Medicine, School of Public Health and Preventive Medicine, Monash University, Melbourne, Australia.

**Table of Content**

[Supplementary Figure 1: Flow chart of study population.	3](#_Toc200373174)

[Supplementary Table 1: STROBE	4](#_Toc200373175)

[Supplementary Table 2: Patient characteristics and final diagnoses comparing patients with and without data on bedside echocardiography (TTE)	6](#_Toc200373176)

[Supplementary Table 3: probable causes of troponin elevations	7](#_Toc200373177)

[Supplementary Table 4: Pathological finding by final diagnosis	8](#_Toc200373178)

[Supplementary Table 5: Regional wall-motion abnormalities in the diagnostic work-up of patients with suspected acute myocardial infarction	9](#_Toc200373179)

Supplementary Figure 1: Flow chart of study population.

Supplementary Table 1: STROBE

|  | Item No | Recommendation | | Page No |  |
| --- | --- | --- | --- | --- | --- |
| **Title and abstract** | 1 | (*a*) Indicate the study’s design with a commonly used term in the title or the abstract | |  |  |
|  |  | (*b*) Provide in the abstract an informative and balanced summary of what was done and what was found | | 1 |  |
| Introduction | | | | | |
| Background/rationale | 2 | Explain the scientific background and rationale for the investigation being reported | | 3 |  |
| Objectives | 3 | State specific objectives, including any prespecified hypotheses | | 3 |  |
| Methods | | | | | |
| Study design | 4 | Present key elements of study design early in the paper | | 4 |  |
| Setting | 5 | Describe the setting, locations, and relevant dates, including periods of recruitment, exposure, follow-up, and data collection | | 4 |  |
| Participants | 6 | (*a*) Give the eligibility criteria, and the sources and methods of selection of participants. Describe methods of follow-up | | 4 |  |
|  |  | (*b*) For matched studies, give matching criteria and number of exposed and unexposed | | n/a |  |
| Variables | 7 | Clearly define all outcomes, exposures, predictors, potential confounders, and effect modifiers. Give diagnostic criteria, if applicable | | 4,5 |  |
| Data sources/ measurement | 8* | For each variable of interest, give sources of data and details of methods of assessment (measurement). Describe comparability of assessment methods if there is more than one group | | 4 |  |
| Bias | 9 | Describe any efforts to address potential sources of bias | | 5 |  |
| Study size | 10 | Explain how the study size was arrived at | | 4 |  |
| Quantitative variables | 11 | Explain how quantitative variables were handled in the analyses. If applicable, describe which groupings were chosen and why | | 5 |  |
| Statistical methods | 12 | (*a*) Describe all statistical methods, including those used to control for confounding | | 5 |  |
|  |  | (*b*) Describe any methods used to examine subgroups and interactions | | 5 |  |
|  |  | (*c*) Explain how missing data were addressed | | 5 |  |
|  |  | (*d*) If applicable, explain how loss to follow-up was addressed | | 4 |  |
|  |  | (*e*) Describe any sensitivity analyses | | n/a |  |
| Results | | |  | |  |
| Participants | 13* | (a) Report numbers of individuals at each stage of study—eg numbers potentially eligible, examined for eligibility, confirmed eligible, included in the study, completing follow-up, and analysed | | 4 & supplement |  |
|  |  | (b) Give reasons for non-participation at each stage | |  |  |
|  |  | (c) Consider use of a flow diagram | | supplement |  |
| Descriptive data | 14* | (a) Give characteristics of study participants (eg demographic, clinical, social) and information on exposures and potential confounders | |  |  |
|  |  | (b) Indicate number of participants with missing data for each variable of interest | |  |  |
|  |  | (c) Summarise follow-up time (eg, average and total amount) | |  |  |
| Outcome data | 15* | Report numbers of outcome events or summary measures over time | | Table 2 |  |

Supplementary Table 2: Patient characteristics and final diagnoses comparing patients with and without data on bedside echocardiography (TTE)

|  | **All (N=3344)** | **without TTE (N=521)** | **with TTE (N=2823)** | **Absolute standardized difference** |
| --- | --- | --- | --- | --- |
| Age (years) | 63.0 (51.0, 75.0) | 65.0 (51.0, 76.0) | 63.0 (51.0, 75.0) | -2 (-5, 0) |
| Males | 2132 (63.8) | 331 (63.5) | 1801 (63.8) | 0.27 (-4.3, 4.9) |
| Body mass index (kg/m²) | 26.0 (24.0, 30.0) | 27.0 (24.0, 30.0) | 26.0 (23.7, 30.0) | -1 (-2, -2) |
| Hypertension | 2119 (63.5) | 333 (64.3) | 1786 (63.4) | -0.91 (-5.5, 3.7) |
| Hypercholesterinemia | 1095 (32.8) | 174 (33.5) | 921 (32.6) | -0.84 (-5.4, 3.7) |
| Smoking, active | 725 (22.2) | 120 (23.4) | 605 (22.0) | -1.44 (-5.5, 2.7) |
| Smoking, former | 729 (22.3) | 124 (24.2) | 605 (22.0) | -2.22 (-6.4, 1.9) |
| Diabetes Mellitus (any type) | 424 (12.8) | 70 (13.6) | 354 (12.6) | -0.95 (-4.3, 2.4) |
| Family history of coronary artery disease | 594 (18.8) | 68 (14.0) | 526 (19.6) | 5.66 (2.1, 9.2) |
| History of stroke | 195 (5.8) | 45 (8.7) | 150 (5.3) | -3.35 (-6, -0.7) |
| Atrial fibrillation | 471 (14.1) | 133 (25.6) | 338 (12.0) | -13.6 (-17.7, -9.5) |
| History of myocardial infarction | 561 (16.8) | 81 (15.6) | 480 (17.0) | 1.45 (-2.1, 5) |
| History of heart failure | 382 (11.4) | 84 (16.2) | 298 (10.6) | -5.6 (-9.1, -2.1) |
| Ischemic signs in first ecg | 664 (20.4) | 109 (22.0) | 555 (20.1) | -1.9 (-6, 2.2) |
| Angiography done | 790 (23.6) | 100 (19.2) | 690 (24.4) | 5.25 (1.4, 9.1) |
| PCI done | 401 (12.0) | 46 (8.8) | 355 (12.6) | 3.75 (0.9, 6.6) |
| eGFR (mL/min for 1.73m²) | 78.1 (59.5, 93.0) | 76.7 (57.6, 93.0) | 78.7 (60.0, 93.1) | 1.99 (-0.8, 4.9) |
| **Discharge diagnosis:** |  |  |  |  |
| NSTEMI Type1 | 281 (8.4) | 28 (5.4) | 253 (9.0) | 3.59 (1.3, 5.9) |
| NSTEMI Type2 | 145 (4.3) | 31 (6.0) | 114 (4.0) | -1.91 (-4.2, 0.4) |
| Acute Injury | 103 (3.1) | 20 (3.8) | 83 (2.9) | -0.9 (-2.8, 1) |
| Chronic injury | 863 (25.8) | 180 (34.5) | 683 (24.2) | -10.35 (-14.8, -5.9) |
| Unstable angina | 256 (7.7) | 20 (3.8) | 236 (8.4) | 4.52 (2.5, 6.6) |
| Stable angina | 26 (0.8) | 2 (0.4) | 24 (0.9) | 0.47 (-0.3, 1.2) |
| Cardiac non-coronary chest pain | 546 (16.3) | 82 (15.7) | 464 (16.4) | 0.7 (-2.8, 4.2) |
| Non-cardiac chest pain | 1124 (33.6) | 158 (30.3) | 966 (34.2) | 3.89 (-0.5, 8.3) |

Median (25^th^, 75^th^ percentile) or n (%)

Supplementary Table 3: probable causes of troponin elevations

| **Non-ST elevation myocardial infarction Type 2** | **N=113** |
| --- | --- |
| Arrythmias (e.g., atrial fibrillation, atrial tachycardia, ventricular tachycardia, bradycardia) | 49 (43.4%) |
| Hypertension | 40 (35.4) |
| Vasospastic angina | 5 (4.4) |
| Non-obstructive coronary artery disease | 7 (6.2) |
| Anemia | 3 (2.7 |
| Respiratory failure | 2 (1.8) |
| Other | 7 (6.2) |
| **Acute myocardial injury** | **N=82** |
| Myocarditis | 11 (13.6) |
| Arrythmias (e.g., atrial fibrillation, atrial tachycardia, ventricular tachycardia, bradycardia) | 5 (6.1%) |
| Takotsubo Cardiomyopathy | 12 (14.8) |
| Heart Failure | 24 (29.6) |
| Hypertension | 2 (2.5) |
| Aortic valve stenosis | 3 (3.7) |
| Pulmonary embolism | 11 (13.6) |
| Pathologies of the aorta | 3 (3.7) |
| Pulmonary infection | 2 (2.5) |
| Non-obstructive coronary artery disease | 1 (1.2) |
| Hypotension | 1 (1.2) |
| Cardiac contusion | 1 (1.2) |
| Sepsis | 1 (1.2) |
| Stroke | 1 (1.2) |
| Other | 3 (3.7) |
| **Chronic myocardial injury** | **N=669** |
| Myocarditis | 6 (0.9) |
| Arrythmias (e.g., atrial fibrillation, atrial tachycardia, bradycardia, premature ventricular contractions) | 62 (9.2) |
| Heart failure | 109 (16.4) |
| Hypertension | 162 (24.4) |
| Aortic valve stenosis | 26 (3.9) |
| Pulmonary embolism | 10 (1.5) |
| Pathologies of the aorta | 4 (0.6) |
| Pulmonary infection | 5 (0.8) |
| Chronic obstructive pulmonary disease and pulmonary hypertension | 10 (1.5) |
| Non-obstructive coronary artery disease | 83 (12.5) |
| Anemia | 5 (0.8) |
| Post PCI | 1 (0.2) |
| Chronic kidney disease | 35 (5.3) |
| Amyloidosis or sarcoidosis | 1 (0.2) |
| Cardiotoxicity (e.g., chemotherapy) | 2 (0.3) |
| Exercise | 1 (0.2) |
| Obstructive coronary artery disease | 114 (17.1) |
| Other | 29 (4.4) |

N (% within each subgroup)

Supplementary Table 4: Pathological finding by final diagnosis

|  | **All (N=2779)** | **no pathological findings (N=2128)** | **any pathological findings (N=651)** |
| --- | --- | --- | --- |
| NSTEMI Type1 No. (%) | 253 (9.1) | 123 (5.8) | 130 (20.0) |
| NSTEMI Type2 No. (%) | 113 (4.1) | 70 (3.3) | 43 (6.6) |
| Acute Injury No. (%) | 82 (3.0) | 40 (1.9) | 42 (6.5) |
| Chronic injury No. (%) | 669 (24.1) | 414 (19.5) | 255 (39.2) |
| Unstable angina pectoris No. (%) | 231 (8.3) | 174 (8.2) | 57 (8.8) |
| Stable angina pectoris No. (%) | 24 (0.9) | 17 (0.8) | 7 (1.1) |
| CNCCP No. (%) | 455 (16.4) | 394 (18.5) | 61 (9.4) |
| NCCP No. (%) | 952 (34.3) | 896 (42.1) | 56 (8.6) |

Supplementary Table 5: Regional wall-motion abnormalities in the diagnostic work-up of patients with suspected acute myocardial infarction

|  | N | Sensitivity | Specificity | PPV | NPV | TP | TN | FN | FP |
| --- | --- | --- | --- | --- | --- | --- | --- | --- | --- |
| All patients | 1636 | 44.5 (38.5, 50.8) | 86.2 (84.3, 88.0) | 36.5 (31.3, 42.1) | 89.7 (88.0, 91.3) | 110 | 1198 | 137 | 191 |
| ESC 0/1-hour Observe | 599 | 37.8 (25.1, 52.4) | 78.9 (75.3, 82.1) | 12.7 (8.1, 19.4) | 94.0 (91.4, 95.8) | 17 | 437 | 28 | 117 |
| ESC 0/1-hour Rule-in | 308 | 47.2 (40.4, 54.2) | 65.8 (56.5, 73.9) | 71.0 (62.7, 78.1 | 41.2 (34.3, 48.6) | 93 | 73 | 104 | 38 |
